# Virtual Reality Telerehabilitation for Postural Instability in Parkinson's Disease: A Multicenter, Single-Blind, Randomized, Controlled Trial

**DOI:** 10.1155/2017/7962826

**Published:** 2017-11-26

**Authors:** Marialuisa Gandolfi, Christian Geroin, Eleonora Dimitrova, Paolo Boldrini, Andreas Waldner, Silvia Bonadiman, Alessandro Picelli, Sara Regazzo, Elena Stirbu, Daniela Primon, Christian Bosello, Aristide Roberto Gravina, Luca Peron, Monica Trevisan, Alberto Carreño Garcia, Alessia Menel, Laura Bloccari, Nicola Valè, Leopold Saltuari, Michele Tinazzi, Nicola Smania

**Affiliations:** ^1^Neuromotor and Cognitive Rehabilitation Research Center (CRRNC), Department of Neurosciences, Biomedicine and Movement Sciences, University of Verona, P.le L.A. Scuro 10, 37134 Verona, Italy; ^2^Neurorehabilitation Unit, Azienda Ospedaliera Universitaria Integrata, P.le Scuro 10, 37134 Verona, Italy; ^3^Department of Rehabilitation Medicine, ULSS2 Marca Trevigiana, Treviso, Italy; ^4^Rehabilitation Hospital of Motta di Livenza, Motta di Livenza, Italy; ^5^Private Clinic Villa Melitta, Neurological Rehabilitation, 39100 Bozen, Italy; ^6^Research Department for Neurorehabilitation South Tyrol, 39100 Bozen, Italy; ^7^School of Specialization in Physical Medicine and Rehabilitation, University of Verona, Verona, Italy; ^8^Azienda ULSS N. 9, Treviso, Italy; ^9^Azienda ULSS N. 15, Rehabilitation Unit, Alta Padovana, Cittadella, Italy; ^10^Azienda ULSS N. 1, Belluno, Italy; ^11^Department of Neurology, Hochzirl Hospital, Zirl, Austria; ^12^Neurology Unit, Movement Disorders Division, Department of Neurosciences, Biomedicine and Movement Sciences, University of Verona, Verona, Italy

## Abstract

**Introduction:**

Telerehabilitation enables patients to access remote rehabilitation services for patient-physiotherapist videoconferencing in their own homes. Home-based virtual reality (VR) balance training has been shown to reduce postural instability in patients with Parkinson's disease (PD). The primary aim was to compare improvements in postural stability after remotely supervised in-home VR balance training and in-clinic sensory integration balance training (SIBT).

**Methods:**

In this multicenter study, 76 PD patients (modified Hoehn and Yahr stages 2.5–3) were randomly assigned to receive either in-home VR telerehabilitation (*n* = 38) or in-clinic SIBT (*n* = 38) in 21 sessions of 50 minutes each, 3 days/week for 7 consecutive weeks. VR telerehabilitation consisted of graded exergames using the Nintendo Wii Fit system; SIBT included exercises to improve postural stability. Patients were evaluated before treatment, after treatment, and at 1-month follow-up.

**Results:**

Analysis revealed significant between-group differences in improvement on the Berg Balance Scale for the VR telerehabilitation group (*p* = 0.04) and significant Time × Group interactions in the Dynamic Gait Index (*p* = 0.04) for the in-clinic group. Both groups showed differences in all outcome measures over time, except for fall frequency. Cost comparison yielded between-group differences in treatment and equipment costs.

**Conclusions:**

VR is a feasible alternative to in-clinic SIBT for reducing postural instability in PD patients having a caregiver.

## 1. Introduction

Up to 75% of people with Parkinson's disease (PD) have impaired postural control, which can often lead to postural instability and an increased risk of falls as a consequence [1]. While postural instability in PD may involve dysfunctions in several subsystems [2, 3], it is the impaired multimodal integration of sensory feedback from the visual, proprioceptive, and vestibular systems that can predominantly contribute to deficits in gait and balance [4, 5] as seen in other neurological conditions [6, 7]. Moreover, since dopaminergic medications have limited effects on postural instability in PD, rehabilitation is perhaps the most effective nonpharmacological approach to reducing the risk of falls [8]. National and international guidelines stress the importance of starting rehabilitation at an early stage of the disease to prevent mobility-related disability and comorbidities (e.g., pain) [9]. However, growing disability, travel distances, and uneven distribution of rehabilitation services can make access to care problematic for PD patients [10].

An innovative approach to fitness training and rehabilitation is exergaming with active video games that combine body movement with gaming skill, in which players use body movements to control and play games in engaging virtual reality (VR) scenarios. An additional advantage of exergaming is that it can be performed independently at home with minimal equipment and at accessible cost. For example, the highly popular Nintendo Wii Fit and balance board is a VR fitness application that holds potential for use as an adjunct to sensory integration balance training (SIBT), one of the most effective ways to improve postural stability in PD [11].

VR-based telerehabilitation balance programs have been proven feasible and effective in several neurological conditions [12, 13]. While telerehabilitation and VR applications have been used separately to evaluate or treat balance dysfunctions in PD [14–18], whether they are feasible, safe, and effective when combined with a telerehabilitation setting warrants investigation. The primary aim of this study was to compare improvements in postural stability after in-home VR-based balance training with the Nintendo Wii Fit system (TeleWii) and after in-clinic SIBT in PD patients. The secondary aim was to compare pre- and posttreatment differences in perceived balance confidence, mobility-related function, quality of life, fall frequency, and the difference in the costs of the two rehabilitation programs. We hypothesized that TeleWii might be as effective as SIBT in improving postural control by acting on sensorimotor integration processes and that the total costs for the in-home TeleWii program would be lower than in-clinic SIBT.

## 2. Methods

Four neurorehabilitation units [Neuromotor and Cognitive Rehabilitation Research Center (CRRNC) AOUI Verona, Azienda ULSS N. 1 Belluno, Azienda ULSS N. 9 Treviso, and Azienda ULSS N. 15 Alta Padovana, Cittadella] participated in this multisite, single-blind randomized controlled trial (RCT). The participating centers, all located in Veneto, deliver rehabilitation services in predominantly rural areas.

### 2.1. Participants

From December 2013 to December 2015, consecutive outpatients with medical diagnosis of PD confirmed according to the United Kingdom Parkinson's Disease Society Brain Bank criteria [19] were assessed for eligibility. Inclusion criteria were age > 18 years, modified Hoehn and Yahr (H&Y) stages 2.5 to 3 [20], stable medication usage in the previous month, ability to perform postural transfer and maintain upright standing posture for at least 10 minutes, and the presence of a caregiver. Exclusion criteria were cardiovascular, orthopedic, and otovestibular disorders (dizziness); visual or other neurological conditions that could interfere with balance; severe dyskinesias or on-off fluctuations; Mini-Mental State Examination (MMSE) score < 24/30; and severe depression as measured on the Geriatric Depression Scale (GDS). All patients were informed about the experimental nature of the study and gave their written informed consent before enrolment. The study was carried out following the tenets of the Declaration of Helsinki and approved by the local ethics committee (n. 75/CE, AULSS9, RSF 319/2010).

### 2.2. Interventions

The trial steering committee designed two treatment protocols. To ensure uniform delivery of treatment, one physiotherapist from each center received instruction in the TeleWii protocol, and one physiotherapist received instruction in the SIBT protocol [11]. Both groups underwent 21 individualized treatment sessions of 50 minutes each, 3 days/week (Monday, Wednesday, and Friday) for 7 consecutive weeks. During the study period, patients were not allowed to receive any other type of rehabilitation. No other restrictions on physical activity were set.

#### 2.2.1. TeleWii Set-Up

A TeleWii-Lab comprising the Nintendo Wii console for motion controlled inputs, the Wii Fit gaming system, and balance board (Nintendo Co., Ltd., Kyoto, Japan) was set up at each rehabilitation unit [21]. For this study, a laptop computer connected to a high-resolution web-camera was used to establish real-time remote visual communication via Skype® software (Skype/Microsoft) between the rehabilitation unit and the patient's home. A research team member installed an identical TeleWii set-up at the patient's home.

#### 2.2.2. TeleWii Training

Patients were instructed to exercise with TeleWii only when in the ON state. The physiotherapist gave a full explanation of the training protocol and conducted a trial TeleWii session at the hospital lab. A logbook was provided for reporting any issues with the Skype connection or difficulties with the training program. In-home TeleWii training consisted of 21 sessions of balance exercises of 50 minutes each. During each session, the physiotherapist supervised two patients simultaneously. A brief warm-up consisted of stretching exercises of the upper and lower extremities with self-applied gentle joint mobilization while lying supine on a mat. TeleWii training included the following 10 exergames selected by the physiotherapist according to the patient's clinical condition and progressive improvement over time [15]. The Skype video calls lasted the entire duration of each training session. A caregiver was always present to monitor the patient during training and warrants its safety (Table 1).

#### 2.2.3. Sensory Integration Balance Training

In-clinic SIBT consisted of 21 sessions of balance and gait exercises lasting 50 minutes each. A brief warm-up session of stretching exercises was followed by static and dynamic balance exercises under different sensory conditions (free vision, blindfolded, wearing a visual-conflict dome, firm/compliant surfaces, and neck extensions) (Table 2) [7, 11, 22].

During each session, the patients performed 10 exercises, at random: 4 self-destabilization, 4 external destabilization, and 2 combined self-destabilization and external destabilization exercises. Each exercise was repeated from 5 to 10 times for 5 minutes depending on the patient's capabilities. Exercises were progressed by increasing the number of repetitions, the task difficulty (greater forward/sideward stepping distance, thicker compliant surface), and the duration of holding a given position. The PT gave verbal and manual instructions and, when necessary, provided support at the patient's pelvis or chest [11].

## 3. Outcomes

At each study center, outcomes were assessed by a single examiner blinded to treatment assignment. Gait and balance measures were evaluated before treatment (T0), after treatment (T1), and at 1-month follow-up (T2). The test order was the same across all evaluation sessions as reported below. Measurements and interventions were conducted with the patients in the ON state.

### 3.1. Primary Outcome

The Berg Balance Scale (BBS) is a 14-item validated scale that evaluates static and dynamic balance dysfunctions (score range 0–56, with higher scores indicating better performance). The minimal detectable change (MDC) is 5 points for PD patients [23].

### 3.2. Secondary Outcomes

The Activities-Specific Balance Confidence (ABC) scale evaluates a patient's perceived level of balance confidence in activities of daily living (score range 0–100, with higher scores indicating better performance) [24]. A score below 75.6 suggests increased risk of falls. The 10-Meter Walking Test (10-MWT) measures gait speed. The minimal clinically important difference (MCID) scores in the geriatric population are 0.05 m/sec (small meaningful change) and 0.13 m/sec (substantial meaningful change) [25]. The minimal detectable change in PD patients is 0.25 m/sec (fastest gait speed) [23]. The Dynamic Gait Index (DGI) evaluates an individual's ability to modify gait in response to task demands (score range 0–24, with higher scores indicating better performance). The MCID for older adults with a DGI score < 21/24 is 1.80 [26]. Parkinson's Disease Quality of Life questionnaire (PDQ-8) measures quality of life [27]. The number of falls in the previous month was recorded in a self-report log. At the follow-up evaluation, the patients completed a satisfaction questionnaire investigating domains considered relevant for the patient; responses for each domain were marked on a 5-point Likert-type scale (1: strongly agree; 5: strongly disagree) (Table 3).

Patients were provided with logbook to record their feelings and any difficulties or adverse events they had experienced at each training session.

#### 3.2.1. Costs of Rehabilitation

The direct cost categories included the cost of personnel for screening, assessments (before, after, and follow-up), treatments (one-session training and treatments), and resource utilization. Personnel costs (in euro) were calculated based on the amount of work an average worker performs in 1 hour (staff-hour approach) according to national standard rates. Costs for resource utilization (per type) were calculated taking into account a depreciation rate of 20% per year of the average market value. Indirect costs (utilities, facilities, etc.) were calculated as 25% of the direct costs according to the Italian manual for costing healthcare in public hospitals.

### 3.3. Sample Size

For sample size calculation, we estimated that 70 patients (35 per group) would provide 90% power (5% probability of type 1 error) to detect a difference pre- and posttreatment of 4.5 points (variance 33.64) on the BBS score (primary outcome) [28]. Assuming a 9% dropout rate, a total of 76 patients were necessary to perform this study.

### 3.4. Randomization

The principal investigator (NS) was responsible for randomization procedures. After screening, a list was generated using computer-generated random number tables (allocation ratio 1 : 1). Eligible patients were consecutively entered into the list and allocated to the TeleWii or the SIBT group.

### 3.5. Statistical Analysis

The single imputation (simple mean) method was used to handle missing data. Descriptive statistics included means and standard deviation. The *X*^2^ test was utilized for categorical variables. Since the data were normally distributed (Shapiro-Wilk Test), parametric tests were used for inferential statistics. A two-way mixed ANOVA was applied using “Time” as the within-group factor and “Group” as the between-group factor. Two-tailed Student's *t*-test for unpaired data was used for between-group comparisons. The clinical relevance of changes in primary and secondary outcome scores after treatment and at follow-up was evaluated according to published MCID values [25, 26]. The level of significance was set at *p* < 0.05. Bonferroni's correction was applied for multiple comparisons (*p* < 0.025). Statistical analysis was performed with SPSS 20.0 (IBM SPSS Statistics for Windows, Version 20.0. Armonk, NY, USA).

## 4. Results

One hundred and thirty-five patients were consecutively assessed to the neurorehabilitation centers. Twenty-six patients were excluded because they did not meet the inclusion criteria, 13 declined to participate in this study, and 20 had technological issue including the lack of Internet connection and motivation of using technology.

**Figure 1:**
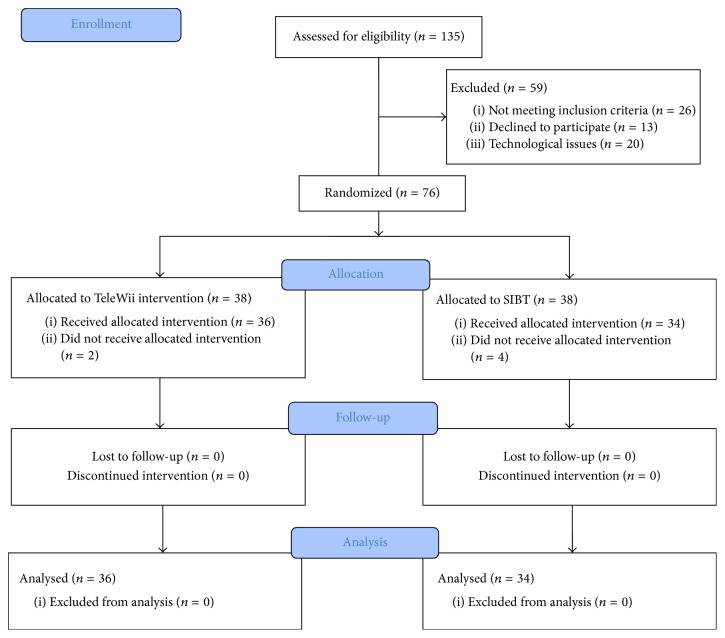
Flow diagram.

A total of 76 patients with idiopathic PD were randomized to the TeleWii (*n* = 38) or the SIBT (*n* = 38) group; 36 in the TeleWii and 34 in the SIBT group completed the study. Two patients in the TeleWii group and 4 in the SIBT group withdrew for medical reasons or because of difficulty arranging transportation to the study site (Figure 1). No adverse events were reported during the study period.

There were no significant between-group differences in demographic and clinical data (Table 4) or in primary and secondary outcome measures at baseline (T0).

### 4.1. Primary Outcome

Significant between-group differences were found for BBS scores (*p* = 0.04) (Table 5). Post hoc between-group comparisons showed that these differences were significant at 7 weeks (completion of training programs [T1]) (*p* = 0.02). Both groups showed an overall significant improvement in performance at T1 and at follow-up evaluation (T2). At T1, the SIBT group improved by 4.21 (*p* < 0.001), and the TeleWii improved by 3.74 (*p* < 0.001). At T2, the SIBT group and the TeleWii improved by 4.05 and 3.21, respectively (Table 5).

### 4.2. Secondary Outcomes

There were no significant between-group differences in secondary outcomes. A significant “Time *∗* Group” interaction was found in the DGI. The difference in the DGI for the SIBT group reached the MCID at T1 but fell below it at T2 (1.71 instead of 1.80). The difference for the TeleWii group was 0.85 at T1 and 0.93 at T2. Both groups showed an overall significant improvement as measured on the ABC, 10-MWT, DGI, and PDQ-8 (Table 5). The difference in the 10-MWT for the SIBT group indicated a substantial change in performance at T1 (0.14) and a small change at T2 (0.05). The difference in the 10-MWT for the TeleWii group was 0.03 at T1 and 0.02 at T2.

There was no statistical significant difference in satisfaction rates between the TeleWii (mean score 4.57 ± 0.32) and the SIBT groups (mean score 4.66 ± 0.32).

The total cost of rehabilitation was €23.299,00 for the TeleWii group and €28.899,80 for the SIBT group. In both groups, the breakdown in total cost per patient was €24 for physiatrist screening and €28.20 for physiotherapy evaluation (posttreatment and follow-up). The initial physiotherapy evaluation cost is €56.40 because an additional session was required (the first part of the two-step procedure). The total treatment cost was €246.75 for the TeleWii group and €493.50 for the SIBT group. The equipment cost was €106.90 for the TeleWii group and €6.30 for the SIBT group. The indirect costs were €122.63 for the TeleWii group and €152.11 for the SIBT group. The total cost for rehabilitation for patient was € 383.55 for the TeleWii group and € 602. 1 for the SIBT group.

## 5. Discussion

Two main findings emerged from this study. First, static and dynamic postural control was improved in the PD patients who had received in-home VR-based balance training (TeleWii), while improvements in mobility and dynamic balance were greater, on average, in those who had received in-clinic SIBT. However, the practical relevance of these differences was minimal. Second, comparable effects on perceived confidence in performing ambulatory activities, gait speed, fall frequency, and quality of life were achieved with both treatment modalities. In addition, the total cost of rehabilitation using TeleWii was lower than that of SIBT.

The Nintendo Wii Fit system has been proposed as a feasible and useful tool for balance training in people with PD [14, 16–18, 28, 29]. Its rationale relies on providing augmented visual and auditory feedback to progressively challenge postural control during a given task. This strategy might bypass the deficient internal motor generation system present in PD patients and improve motor response [30]. Two studies have examined the effects of balance training on postural instability in PD using a home-based setting [16, 28]. In a study published by Esculier et al. [16] involving 10 patients with moderate PD and 8 healthy controls, patients received 6-week home-based balance training using Nintendo Wii and balance board (40 min/session, 3 sessions/week). At the end of the treatment, the PD group reported significant improvements in static and dynamic balance, mobility, and functional ability when compared with healthy controls. Although no significant within-group changes were reported on the ABC scale, at the end of the program the PD patients reported increased balance and stability in activities of daily living (ADL). Zalecki and colleagues [28] reported similar findings in a larger sample of PD patients (*n* = 24) with moderate PD in which scores on the BBS scale were improved by 4.5 points and by 6.5 points on the ABC scale after treatment. The lack of a control group of patients and follow-up evaluation precludes drawing any conclusions, however. Nevertheless, these positive findings warrant future study.

Exergaming with Nintendo Wii has been shown to improve static and dynamic postural control in people with PD as evaluated on the BBS [16, 28]. In our study, a statistically significant difference was found for the TeleWii group. Though neither training modality achieved a MCID (5 points) in the BBS [23], the improvement was greater after SIBT than TeleWii (4.21 versus 3.74 points).

In both groups, the training effects may be ascribed to the improved use of different resources for postural stability and orientation [2]. First, the improvement in postural reactions and movement strategies (i.e., reactive, anticipatory, and voluntary) may have been related to the different training modalities. The SIBT protocol involves more dynamic training, whereas the TeleWii requires the use of feedback, feedforward, and voluntary strategies while performing the exergames, which are quasi-static and focused mainly on self-destabilization tasks. The balance training offered by TeleWii consisted of exercises such as weight shifting, symmetric foot stepping, and controlled movements near the limits of stability repeated in a high number of repetitions and a complex and motivating environment. All these tasks required an active control of body alignment and tone concerning gravity, support surface, visual environment, and internal references. Based on the interpretation of convergent sensory information from somatosensory, vestibular, and visual systems, the patient was requested to implement anticipatory postural adjustments to stabilize the body's center of mass and select an appropriate motor sequence to accomplish the task. In contrast, the lack of exercises focused on compensatory postural adjustments induced by external destabilization should be acknowledged as the main drawback of this approach. The execution of external destabilization exercises could be included in such rehabilitation protocols only with a greater involvement of the caregiver. The effects of TeleWii training may have been reinforced by visual and auditory cueing, which in our study was conceptualized as a motor-learning tool, and by feedback on balance performance that motivated patients to make appropriate postural adjustments [29].

Second, it is conceivable that Wii training led to improvements in sensory strategies (i.e., sensorimotor integration and reweighting). With progressive training, patients were able to rapidly reweight and select the more reliable sensory information to maintain their postural stability. Although postural instability may have multifactorial causes, it primarily results from impaired central integration [3, 31]. In our previous studies, we showed that SIBT might improve sensory integration processes not only in PD [11] but also in other neurological diseases [6, 7]. Similarly, TeleWii might improve the ability to integrate and reweight the incoming sensory inputs and shape the system of coordinates on which the body's postural control is based [2]. In addition, it offers an enriched VR environment of visual and auditory cueing that may improve motor learning [15, 30].

Finally, VR-based exercise programs have been shown to elicit the integration of motor and cognitive abilities (i.e., attention, executive functions) and stimulate the brain's reward circuitry [16, 28, 30, 32]. VR engages participants in cognitive and motor activities (i.e., dual tasking) simultaneously that require planning, attention, sensory integration, and processing of stimuli from the virtual environment [30]. TeleWii enhances this experience more than SIBT by its ability to deliver a combined motor-cognitive experience in an ecologically valid therapeutic environment [29, 30].

SIBT was found to be more effective than TeleWii on the DGI, reaching a higher MCID score than TeleWii after training. This is particularly relevant, given that postural instability and falls [33] in PD become a clinical concern in the middle stages of the illness, though walking difficulties and unsteadiness while turning may often arise also in the early stage of PD. The SIBT training effects may also be ascribed to gain in strength and lean body mass. Although we did not include any measure of muscle strength and body mass among outcome measures, it is conceivable that the exercises requiring postural transfers and walking (for detail see Table 2) may lead to gains in strength and lean body mass. Future studies should consider the training effects of both pieces of training regarding biomechanical constraints that may affect balance such as muscle strength and lean body mass [2].

TeleWii opens new opportunities for treating postural instability, giving individuals access of care from their home [10] especially for those residing in rural areas. This model saves time and travel costs and allows the delivery of rehabilitation services at scale (i.e., one physiotherapist monitoring two or more patients). In our study, this approach was the main factor that reduced the treatment cost, whereby one physiotherapist supervised two patients in real-time. Second, competent staff can supervise training to address specific deficits and adjust task complexity accordingly [10].

The strengths of the present study are the large patient sample and the comprehensive evaluation of balance disorders about different functions and domains. Its limitations are the lack of instrumental evaluation to assess balance performance, postural reactions, and changing muscle strength and lean body mass. Moreover, these findings cannot be generalized to PD patients with significant cognitive decline, because the use of TeleWii may be unsafe.

To conclude, as a part of the multifaceted management of motor symptoms in PD, TeleWii is a feasible and valid alternative to SIBT for reducing postural instability in PD patients at modified Hoehn and Yahr stages 2.5–3 and having caregiver assistance. TeleWii holds promise and potential to enrich rehabilitation care at home in people with PD but policy issues, especially reimbursement, need still to be addressed.

## Figures and Tables

**Table 1:** TeleWii balance training program.

Name of exercise	Exercise description	Expected impact on mobility
Table tilt	Shift the body weight in all directions with feet placed in a fixed position. Make a plan of movements to tilt the virtual platform, bring the balls in the holes and go to the next level of difficulty	Improve the correct use of ankle and hip strategy during static condition. Improve the quick change of strategy from ankle to hip and vice versa
Penguin slide	Shift the body weight toward the right and left direction to bend the virtual ice platform, with feet placed in a fixed position. Make a plan of movements to catch as much fishes as possible	Improve the correct use of ankle and especially hip strategy during static condition
Balance bubble	Shift the body weight forward to move the avatar forward; lean left and right to steer	Improve the correct use of ankle and hip strategy during static condition. Improve the quick change of strategy from ankle to hip and vice versa
Ski slalom	Lean the body left and right to ski down a slalom course and pass between flags with your avatar	Improve the correct use of ankle and hip strategy during static condition. Improve the quick change of strategy from ankle to hip and vice versa. Improve the ability to orientate the trunk in the space
Skateboarding	Push off the ground with right/left foot to skate forward with your avatar, and lean left or right to turn; if the speed slows down, lean toward one side to pick up speed; jump off the virtual ramps by raising heels to perform a trick in midair	Improve the correct use of ankle, hip, and stepping strategy during quasi-static condition. Improve the quick change of strategy from ankle to hip or stepping and vice versa. Improve the ability to orientate the trunk in the space
Perfect 10	Shake hips back, front left, or right to add up to the given number with your avatar. The aim is to make a sum of 10	Improve the correct use of ankle and hip strategy during static condition. Improve the quick change of strategy from ankle to hip and vice versa. Improve the dual task performance (motor & cognitive task)
Tilt city	Tilt the Wii remote to move the virtual board at the top of the screen. Shift your body weight left and right to tilt the virtual boards at the bottom. Make a plan of movements to drop the balls into the matching colored pipe	Improve the correct use of hip strategy during static condition. Improve the coordination between upper and lower limbs (dual motor task)
Snowball fight	Shift your body weight right or left to move out your avatar from behind a protective barrier; use the Wii remote at the screen to throw snowballs; when throwing, watch out for incoming snowballs and avoid them by shifting your body weight	Improve the correct use of ankle and hip strategy during static condition. Improve the quick change of strategy from ankle to hip and vice versa. Improve the coordination between upper and lower limbs (dual motor task). Improve the attentional strategies to multiple stimuli
Rhythm parade	Stepping in place to move your avatar and wave the controller when scrolling icons coming from the top of the screen hit the circles place at the bottom	Improve the correct use of all strategies during static condition. Improve the quick change of strategy from hip to stepping and vice versa. Improve the coordination between upper and lower limbs (dual motor task)
Bird's-eye bulls-eye	Stand on the board with feet placed in a fixed position. Flap the arms to land your avatar on the targets; lean in and flap to fly your avatar forward; stay centered on the board and flap to go higher with your avatar; shift the body weight right or left to turn; stop flapping to land your avatar on a target and to get a bonus; rack up bonus time and head for the finish; small flaps help hover; big flaps help to soar	Improve the correct use of ankle and hip strategy during static condition. Improve the quick change of strategy from ankle to hip and vice versa. Improve the coordination between upper and lower limbs, and between upper limbs (dual motor task)

CoM, center of mass; CoP, center of pressure. The exercises are listed in order of task difficulty starting from single-task through dual-task performance.

**Table 2:** Sensory integration balance training program.

Type of exercise	Task explanation	Expected impact
Self-destabilization exercises (mainly feedforward)		
Static weight bearing	In stance with feet placed shoulder-width apart, transfer the body weight back and forth on the tips of the toes and the heels.^*∗*°@∧^	Improve correct use of ankle strategy during static condition.
	In stance with feet placed shoulder-width apart, transfer the body weight mediolaterally from the right to the left foot.^*∗*°@∧^	Improve correct use of ankle and hip strategy during static condition; improve quick change of strategy from ankle to hip and vice versa.
	In stance with feet placed shoulder-width apart, transfer the body weight in all directions (i.e., drawing a cone with head).^*∗*°@∧^	Improve correct use of ankle and hip strategy during static condition; improve quick change of strategy from ankle to hip and vice versa.
Trunk twist	Sitting in a chair without armrests, with feet placed shoulder-width apart on the floor, twist the torso as much as possible toward the right and the left.^*∗*°@^	Improve trunk mobility in sitting conditions.
	In stance with feet placed shoulder-width apart, twist the torso as much as possible toward the right and the left.^*∗*°@∧^	Improve trunk mobility in standing conditions.
Postural transfers	Sitting in a chair without armrests, with feet placed shoulder-width apart on the floor, sit-to-stand.^*∗*°@∧^	Improve correct use of ankle and hip strategy during postural transfers.
	Sitting in a chair without armrests, with feet placed shoulder-width apart on the floor, sit-to-stand while grasping a glass of water.^*∗*°∧^	Improve correct use of ankle and hip strategy during postural transfers; improve coordination between upper and lower limbs (dual motor tasking).
Dynamic weight bearing	In stance with feet placed shoulder-width apart, step up and down in place, varying the height with each step while catching and throwing a ball.^*∗*^	Improve correct use of ankle, hip, and stepping strategy during static condition; improve quick change of strategy from ankle to hip (or stepping) and vice versa; improve coordination between upper and lower limbs (dual motor tasking).
	Front and side lunges.^*∗*°^	Improve correct use of all strategies during dynamic condition; improve quick change of strategy from hip to stepping and vice versa.

External destabilization exercises (mainly feedback)		
External perturbed	In stance with feet placed shoulder-width apart on the floor, recover balance after external perturbations by the PT to the patients' chest/upper back/shoulders in anteroposterior and mediolateral directions.^*∗*°@∧^	Improve correct use of all strategies during quasi-static condition; improve quick change of strategy; improve proper reaction to unexpected postural destabilization in all directions.
Unstable surfaces	In stance work on progressively thicker compliant surfaces (1.5, 3.5, and 8 cm) according to patient's abilities.^*∗*°@+^	Improve correct use of ankle, hip, and stepping strategy during static condition; improve quick change of strategy; improve ability to orientate the trunk in space.
	In an upright position, recover balance on a rigid, square-shaped wooden platform with a roller surface.^*∗*°^	Improve correct use of ankle, hip, and stepping strategy during dynamic conditions; improve quick change of strategy, improve weight bearing ability and capacity to properly orientate the trunk in space.
	Walking over progressively thicker compliant surfaces (1.5, 3.5, and 8 cm) according to patient's abilities.^*∗*°^	Improve correct use of ankle, hip, and stepping strategy during dynamic conditions; improve quick change of strategy; improve weight bearing ability and capacity to properly orientate the trunk in space.
Swiss ball	Maintain balance while sitting on a Swiss ball, with feet placed shoulder-width apart; in the second part of the exercise, the patient alternatively raises the right and the left leg from the floor.^*∗*°^	Improve trunk control, orientation, and stability.

Self-destabilization and external destabilization exercises (feedback and feedforward)		
Dual-task	Keep walking while catching and throwing a ball with the PT.^*∗*^	Improve correct use of all strategies during dynamic condition; improve quick change of strategy; improve proper reaction to unexpected postural destabilization in all directions.
	Keep walking while quickly changing direction (forward, backward, sideways).^*∗*^	Improve correct use of all strategies during dynamic condition; improve quick change of strategy.
	Keep walking while bouncing a ball and switching from right to left hand.^*∗*^	Improve correct use of all strategies during dynamic condition; improve quick change of strategy; improve proper reaction to unexpected postural destabilization in all directions.
	Keep walking while increasing the amplitude of leg movements (increasing stride length) and swing movement of the arms.^*∗*^	Improve correct use of ankle, hip, and stepping strategy during dynamic conditions; improve quick change of strategy; improve coordination between upper and lower limbs (dual motor tasking).
	Keep walking while paddling with a stick.^*∗*^	Improve correct use of ankle, hip, and stepping strategy during dynamic conditions; improve quick change of strategy; improve coordination between upper and lower limbs (dual motor tasking).

CoM, center of mass; CoP, center of pressure; PT, physiotherapist; manipulation of sensory conditions: ^*∗*^free vision, °blindfolded, ^@^wearing a visual-conflict dome, ^∧^firm/compliant surfaces (1.5, 3.5, and 8 cm thick), and ^+^neck extension.

**Table 3:** Satisfaction questionnaire items.

(1) My privacy was respected during my rehabilitation care.
(2) The instructions my physiotherapist gave me were helpful.
(3) All staff members were courteous.
(4) The rehabilitation sessions were carried out on time without delays.
(5) I was satisfied with the number and duration of treatment sessions.
(6) The location of the facility was easily accessible.
(7) My physiotherapist seemed to have a genuine interest in me as a person.
(8) All staff members understood my problem or condition.
(9) I was satisfied with the treatment provided by my physiotherapist.
(10) I was satisfied with the outcomes of rehabilitative treatment.
(11) I was satisfied with the modalities of rehabilitative treatment.
(12) I believe that this type of treatment is adequate to improve my balance disturbances.
(13) I was satisfied with the overall quality of my rehabilitation care.
(14) I would repeat this treatment if I need rehabilitation care in the future.

Responses were scored on a 5-point Likert-type scale from 1 “strongly agree” to 5 “strongly disagree.”

**Table 4:** Baseline demographic and clinical characteristics.

Characteristic	TeleWii Group (*n* = 38)	SIBT group (*n* = 38)	Baseline comparison *p* value
Age (years) (mean ± SD)	67.45 (7.18)	69.84 (9.41)	0.14
Gender (number of males/females)	23/15	28/10	0.22
Disease duration (years) (mean ± SD)	6.16 (3.81)	7.47 (3.90)	0.14
Dominant PD phenotype (NT/T/YO)	21/12/5	14/15/9	0.24
More affected side (B/R/L)	7/21/10	8/20/10	0.95
Modified H&Y stage median (Q1–Q3)	2.50 (2.5–2.5)	2.50 (2.5–3.0)	0.76
UPDRS score (mean ± SD)	44.13 (24.05)	50.76 (24.12)	0.15
Falls (number) (mean ± SD)	0.58 (1.44)	1.84 (5.29)	0.24
MMSE score	26.77 (1.48)	28.64 (6.96)	0.16
GDS score	8.26 (5.17)	9.79 (5.34)	0.21

SD, standard deviation; PD, Parkinson's disease; NT, nontremor dominant; T, tremor dominant; YO, younger onset; B, bilateral; R, right; L, left; Q1: lower quartiles in degrees; Q3: upper quartiles in degrees; H&Y, Hoehn and Yahr; UPDRS, Unified Parkinson's Disease Rating Scale; Falls, number of falls in previous month; MMSE, Mini-Mental State Examination; GDS, Geriatric Depression Scale; *p* < 0.05.

**Table 5:** Descriptive and inferential statistics for clinical outcome measures.

Outcomes	Before T0		After T1		Follow-up T2		Intervention phase		Repeated-measures ANOVA			Post hoc analysis					
	Mean (SD)		Mean (SD)		Mean (SD)		Between-group difference (95% CI) mean (LB, UB)		Group	Time	Time × group	Between-group differences		Within-group differences			
														TeleWii		SIBT	
	TeleWii	SIBT	TeleWii	SIBT	TeleWii	SIBT	After	FU	*p*	*p*	*p*	After *p*	FU *p*	After *p*	FU *p*	After *p*	FU *p*
*Primary outcome*																	
BBS (0–56)	48.63 (6.31)	45.61 (7.97)	52.37 (3.29)	49.82 (5.70)	51.84 (4.53)	49.66 (6.59)	2.54 (0.41, 4.67)	2.18 (−0.40, 4.77)	0.04^*∗*^	<0.001^*∗*^	n.s.	0.02^*∗*^	n.s	<0.001^*∗*^	0.002^*∗*^	<0.001^*∗*^	<0.001^*∗*^
*Secondary outcomes*																	
ABC (0–100)	70.31 (18.17)	64.12 (21.37)	79.62 (14.16)	72.52 (21.20)	76.34 (15.98)	71.73 (19.92)	7.10 (−1.16, 15.36)	4.61 (−3.65, 12.87)	n.s.	<0.001^*∗*^	n.s.	n.s.	n.s.	<0.001^*∗*^	<0.001^*∗*^	<0.001^*∗*^	<0.001^*∗*^
10-MW (m/s)	1.59 (0.49)	1.46 (0.42)	1.62 (0.43)	1.60 (0.44)	1.57 (0.42)	1.52 (0.37)	0.35 (-0.16, 0.23)	0.04 (−0.14, 0.22)	n.s.	0.02^*∗*^	n.s.	n.s.	n.s.	n.s	n.s.	0.035	n.s
DGI	20.39 (2.56)	19.34 (2.49)	21.24 (2.56)	21.18 (2.15)	21.32 (2.81)	21.05 (2.54)	0.53 (−1.03, 1.13)	0.26 (−0.96, 1.49)	n.s.	<0.001^*∗*^	0.04^*∗*^	n.s.	n.s.	0.005^*∗*^	0.008^*∗*^	<0.001^*∗*^	<0.001^*∗*^
Falls (number)	0.58 (1.44)	1.84 (5.30)	0.38 (1.33)	0.61 (1.81)	0.29 (0.94)	0.81 (3.31)	−0.23 (−0.95, 0.49)	−0.52 (−1.65, 0.60)	n.s.	n.s	n.s	n.s.	n.s.	n.s.	0.034^*∗*^	n.s.	n.s.
PDQ-8	30.72 (15.54)	30.53 (16.04)	24.16 (14.78)	24.21 (15.85)	25,82 (14.89)	23.91 (13.20)	−0.05 (−7.06, 6.95)	1.90 (−4.52, 8.34)	n.s.	<0.001^*∗*^	n.s.	n.s.	n.s.	<0.001^*∗*^	0.01^*∗*^	0.016^*∗*^	0.006^*∗*^

Before: pretreatment; after: posttreatment; FU: one-month follow-up; SD: standard deviation; TeleWii: telerehabilitation using virtual reality-based training; SIBT sensory integration balance training; *p*: *p* value; BBS: Berg Balance Scale (higher score indicates better performance); falls, number of falls in the previous month; ABC: Activities Balance Confidence scale (higher score indicates better performance); 10-MWT, 10-Meter Walking Test; DGI, Dynamic Gait Index; PDQ-8, Parkinson's Disease Quality of Life questionnaire; CI: confidence interval; LB: lower bound; UB: upper bound; ANOVA: analysis of variance; ^*∗*^statistically significant. For repeated-measures ANOVA, *p* value is significant if <0.05. For post hoc analysis, *p* is significant if <0.025.
